# Worldwide trends in the burden of asthma symptoms in school-aged children: Global Asthma Network Phase I cross-sectional study

**DOI:** 10.1016/S0140-6736(21)01450-1

**Published:** 2021-10-30

**Authors:** M Innes Asher, Charlotte E Rutter, K Bissell, Chen-Yuan Chiang, Asma El Sony, Eamon Ellwood, Philippa Ellwood, Luis García-Marcos, Guy B Marks, Eva Morales, Kevin Mortimer, Virginia Pérez-Fernández, Steven Robertson, Richard J Silverwood, David P Strachan, Neil Pearce, Global Asthma Network Phase I Study Group, Karen Bissell, Chen-Yuan Chiang, Eamon Ellwood, Philippa Ellwood, Guy B Marks, Refiloe Masekela, Eva Morales, Kevin Mortimer, Neil Pearce, David Strachan, Philippa Ellwood, Eamon Ellwood, Antonela Martinez-Torres, Eva Morales, Virginia Pérez-Fernández, Neil Pearce, Stephen Robertson, Charlotte Rutter, Richard Silverwood, David Strachan, Javier Mallol, Manuel Soto-Martínez, Angelita Cabrera Aguilar, Konstantinos Douros, Mohammed Sabir, Meenu Singh, Virendra Singh, Thevaruparambil Unny Sukumaran, Shally Awasthi, Sushil Kumar Kabra, Sundeep Salvi, Roberto García-Almaráz, J. Valente Mérida-Palacio, Blanca E Del Río Navarro, Sandra Nora González-Díaz, Elsy Maureen Navarrete-Rodriguez, José Félix Sánchez, Adegoke G Falade, Heather J Zar, Angel López-Silvarrey Varela, Carlos González Díaz, Magde Nour, Gazal Dib, Yousser Mohammad, Jing-Long Huang, Sasawan Chinratanapisit, Manuel E Soto-Quirós, Pakit Vichyanond, Pedro Aguilar, Sergio Barba, Lata Kumar, S K Sharma, Francisco J Linares-Zapién, Babatunde O Onadeko, Omer Abdel Aziz Musa, Viviana Aguirre, Manuel Baeza-Bacab, Samira Mohammad, Eliana Cortez, Christina H Gratziou, Kamlesh Chopra, Neeta Milind Hanumante, Hugo Nelson, Alfonso Delgado Rubio, Kue-Hsiung Hsieh, Jayant Shah

**Affiliations:** a Department of Paediatrics: Child and Youth Health, Faculty of Medical and Health Sciences, University of Auckland, Auckland, New Zealand; b School of Population Health, Faculty of Medical and Health Sciences, University of Auckland, Auckland, New Zealand; c Department of Medical Statistics, London School of Hygiene & Tropical Medicine, London, UK; d International Union Against Tuberculosis and Lung Disease, Paris, France; e Division of Pulmonary Medicine, Department of Internal Medicine, Wan Fang Hospital, and Division of Pulmonary Medicine, Department of Internal Medicine, School of Medicine, College of Medicine, Taipei Medical University, Taipei, Taiwan; f Epidemiological Laboratory for Public Health, Research and Development, Khartoum, Sudan; g Paediatric Allergy and Pulmonology Units, Virgen de la Arrixaca University Children‘s Hospital, University of Murcia and IMIB Bio-health Research Institute, Murcia, Spain; h ARADyAL Allergy Network, Murcia, Spain; i Respiratory & Environmental Epidemiology, University of New South Wales, Sydney, NSW, Australia; j Department of Public Health Sciences, University of Murcia, and IMIB Bio-health Research Institute, Murcia, Spain; k Department of Paediatrics, University of Murcia, and IMIB Bio-health Research Institute, Murcia, Spain; l Department of Clinical Sciences, Liverpool School of Tropical Medicine, Liverpool, UK; m Centre for Longitudinal Studies, UCL Social Research Institute, University College London, London, UK; n Population Health Research Institute, St George's, University of London, London, UK

## Abstract

**Background:**

Asthma is the most common chronic disease in children globally. The Global Asthma Network (GAN) Phase I study aimed to determine if the worldwide burden of asthma symptoms is changing.

**Methods:**

This updated cross-sectional study used the same methods as the International study of Asthma and Allergies in Childhood (ISAAC) Phase III. Asthma symptoms were assessed from centres that completed GAN Phase I and ISAAC Phase I (1993–95), ISAAC Phase III (2001–03), or both. We included individuals from two age groups (children aged 6–7 years and adolescents aged 13–14 years) who self-completed written questionnaires at school. We estimated the 10-year rate of change in prevalence of current wheeze, severe asthma symptoms, ever having asthma, exercise wheeze, and night cough (defined by core questions in the questionnaire) for each centre, and we estimated trends across world regions and income levels using mixed-effects linear regression models with region and country income level as confounders.

**Findings:**

Overall, 119 795 participants from 27 centres in 14 countries were included: 74 361 adolescents (response rate 90%) and 45 434 children (response rate 79%). About one in ten individuals of both age groups had wheeze in the preceding year, of whom almost half had severe symptoms. Most centres showed a change in prevalence of 2 SE or more between ISAAC Phase III to GAN Phase I. Over the 27-year period (1993–2020), adolescents showed a significant decrease in percentage point prevalence per decade in severe asthma symptoms (–0·37, 95% CI –0·69 to –0·04) and an increase in ever having asthma (1·25, 0·67 to 1·83) and night cough (4·25, 3·06 to 5·44), which was also found in children (3·21, 1·80 to 4·62). The prevalence of current wheeze decreased in low-income countries (–1·37, –2·47 to –0·27], in children and –1·67, –2·70 to –0·64, in adolescents) and increased in lower-middle-income countries (1·99, 0·33 to 3·66, in children and 1·69, 0·13 to 3·25, in adolescents), but it was stable in upper-middle-income and high-income countries.

**Interpretation:**

Trends in prevalence and severity of asthma symptoms over the past three decades varied by age group, country income, region, and centre. The high worldwide burden of severe asthma symptoms would be mitigated by enabling access to effective therapies for asthma.

**Funding:**

International Union Against Tuberculosis and Lung Disease, Boehringer Ingelheim New Zealand, AstraZeneca Educational Grant, National Institute for Health Research, UK Medical Research Council, European Research Council, and Instituto de Salud Carlos III.

## Introduction

Asthma is the most common non-communicable disease in children and one of the most common chronic diseases in adulthood.^1^, ^2^ It is a major global health problem, with estimated 495 100 deaths from asthma in 2017,^3^ and 22·8 million disability-adjusted life-years in 2017.^4^ More than 1000 asthma deaths each day are similar to the number of deaths from malaria.^5^ Cross-sectional comparisons of the prevalence of asthma in populations require standardised methods that can be implemented in a wide range of settings, and the International Study of Asthma and Allergies in Childhood (ISAAC) is the only worldwide study to achieve this.^6^ ISAAC Phase I (1993–95), repeated in Phase III (2001–03), identified that the prevalence of asthma symptoms (current wheeze) in school-aged children (aged 6–7 years and 13–14 years) was rising in some low-income and middle-income countries.^7^, ^8^

The Global Asthma Network (GAN) was established in 2012, building on the success of ISAAC and incorporating a new collaboration with the International Union Against Tuberculosis and Lung Disease. One of GAN's core activities, GAN Phase I (building on ISAAC and using an identical approach and methods)^9^ includes global surveillance of prevalence and severity of asthma symptoms,^10^ making it the only source of new population-based data on worldwide trends in prevalence of asthma symptoms directly comparable to data from ISAAC Phases I and III. Public health interventions need to be based on science, with up-to-date evidence of the size and trends of health issues.

**Research in context**

**Evidence before this study**

We searched PubMed, Web of Science, and Scopus on March 2, 2020, for papers in English published from Jan 1, 1990, to Dec 31, 2019. The search terms used were “asthma or asthma prevalence”, “children or adolescent”, “cross-sectional”, “time trend or repeated”, “global or worldwide or international”, with the search narrowed subsequently with the search terms “review or meta-analysis” added. A recent review of worldwide trends in asthma prevalence found that the International Study of Asthma and Allergies in Childhood (ISAAC) was the only global study providing direct evidence on changes in prevalence of asthma in school-aged children.

**Added value of this study**

To our knowledge, the Global Asthma Network Phase I study, adopting the same methods as ISAAC, produced the first comparable estimates of prevalence and severity of asthma symptoms in school-aged children over nearly three decades (1993–2020), from 27 centres in 14 countries in the Africa and Eastern Mediterranean, America, Europe, and South-East Asia and Western Pacific regions.

**Implications of all the available evidence**

Asthma is a major worldwide public health problem in school-aged children. Implementing strategies to reduce the prevalence and severity of asthma symptoms (and the associated avoidable asthma morbidity and mortality and subsequent burden of related diseases, including chronic obstructive pulmonary disease) should be a priority for the 21st century.

We hypothesised that globally, the burden (prevalence and severity) of asthma symptoms is changing in school-aged children worldwide. The aim was to conduct asthma symptom surveillance around the world in two age groups of school pupils to examine time trends in prevalence and severity of asthma symptoms from centres that completed GAN Phase I and ISAAC Phase I, Phase III, or both.^10^

## Methods

### Study design and participants

ISAAC Phase I and Phase III were multicentre, multi-country, cross-sectional, population studies in school-aged children, following standardised methods that have been well described^11^, ^12^ and are highly replicable.^13^ This enabled comparisons of prevalence and severity of asthma symptoms between ISAAC Phase I and Phase III and GAN Phase I. Centres that completed GAN Phase I and ISAAC Phase I, Phase III, or both were the study centres for this analysis, and key personnel at the GAN Global Centre (Auckland, New Zealand) were the same throughout ISAAC Phase I, ISAAC Phase III, and GAN Phase I.

Each GAN Phase I investigator completed a registration document and followed the GAN manual.^14^ They gained approval from their local ethics committee and replicated the methods that had been used in their centres for ISAAC; this was documented in the centre report,^14^ which enabled checks to ensure the use of the same geographical sampling frame, sample size, age groups, method of selecting pupils, time of year for data collection, and translations as had been used in ISAAC.^15^ From the sampling frame, ten or more schools were selected at random (or all schools if fewer than ten). The GAN Global Centre checked each centre report with the investigator for validity.

As in ISAAC, the compulsory age group was individuals aged 13–14 years (adolescents) who self-completed written questionnaires at school. The inclusion of individuals aged 6–7 years (children) was optional, and their questionnaires were completed at home by their parents or caregivers. All students of the specified age within schools were included and selected by grade, level, or year or by chronological age. Local ethics committees determined the method of consent (either passive or written) from parents or caregivers of both age groups; however, GAN recommended passive consent as written consent could reduce response rate,^16^ and the adolescents agreed by participating. Additionally, GAN Phase I included questionnaires completed by the parents about themselves, not reported here.

### Procedures

We used seven core written asthma questions for comparisons in this Article. Current wheeze was defined as a positive answer to the question *“*have you (has this child) had wheezing or whistling in the chest in the past 12 months?” Severe asthma symptoms were defined as participants with current wheeze who, in the preceding 12 months, had four or more attacks of wheeze, one or more nights per week with sleep disturbance from wheeze, or wheeze affecting their speech.^17^ Ever having asthma (asthma ever) was defined as a positive answer to the question ‘‘have you (has this child) ever had asthma?’’ Exercise wheeze was defined by a positive answer to the question “in the past 12 months, has your (has this child's) chest sounded wheezy during or after exercise?” And lastly, night cough was defined as a positive answer to the question “in the past 12 months have you (has this child) had a dry cough at night, apart from associated with a cold or chest infection?”

Data and the centre report from each participating centre were submitted to the GAN Global Centre, and quality control checks were completed. Centres with minor deviations from the methods were included in analyses, and these deviations are specified in footnotes in the tables, as in ISAAC.^18^ The data were then transferred to one of two designated GAN Phase I data centres for checking and analysis: Murcia (Spain) for Spanish-speaking and Portuguese-speaking centres and London (UK) for all other languages. A uniform approach to data processing, checking, and analysis was developed, using Stata versions 13–15.^19^

The mean date of questionnaire completion in each centre was used, rather than the year alone as had occurred in ISAAC time-trend studies.^7^, ^8^ High levels of participation were required for inclusion because absent school pupils might be away from school due to asthma symptoms: response rate was required to be at least 80% for adolescents and 70% for children.^9^, ^11^, ^18^ The response rate was defined as the number of core asthma symptom questionnaires returned with at least some symptom data, divided by the number of pupils in the age group. Three of the centre datasets received (one for adolescents and two for children) were excluded from the analyses due to poor (<50%) response rates.

In each centre, estimates of prevalence of symptoms for each age group were obtained by dividing the number of positive responses to each question by the number of completed questionnaires. If apparent inconsistencies were found between responses to a main question and a branched question (one dependent on the response to a main question), these were accepted and not recoded. For regional and global summaries, data for each centre were weighted by the sample size.

We obtained country income categories from the World Bank, with countries categorised into low-income, lower-middle-income, upper-middle-income, and high-income countries.^20^ The gross national income 2001 classification was used, because it was close to the timing of ISAAC Phase III, from which most data were available. Countries were allocated to four regions corresponding to WHO regions of the world, with South-East Asia and Western Pacific regions combined and Africa and Eastern Mediterranean regions combined, because of the small number of centres in these regions. WHO regions and country income category are not synonymous.

### Outcomes and statistical analysis

We sought a sample size of 3000 participants for each age group (with a minimum of 1000 deemed acceptable), a stringent requirement because of the number of hypotheses being tested.

We examined changes in prevalence over time from ISAAC Phase I to GAN Phase I. We calculated the 10-year change in prevalence of symptoms for each centre using the difference between the two timepoints (eg, GAN Phase I and ISAAC Phase III) divided by the number of decades between the mean data collection dates of those timepoints.

We derived estimates of the absolute 10-year rate of change in current wheeze, severe asthma symptoms, ever having asthma, exercise wheeze, and night cough for each centre. We calculated the SE of this change to account for school-level clustering.^19^ We used Bland-Altman plots to examine the relationship between change in prevalence and average prevalence between timepoints, to remove the influence of sampling error (regression to the mean)^21^ when comparing the trends in higher or lower prevalence areas. The relationship was assessed with Spearman's rank correlation.

To examine trends over time in different types of countries or centres, multilevel modelling was undertaken. Along with GAN Phase I centres who participated in ISAAC, we included ISAAC-only centres with data from both ISAAC Phases I and III^7^ (94 centres with adolescents and 57 centres with children). This enabled us to estimate trends in prevalence across the full 27-year period (1993–2020) using all available time trends of centres in one mixed-effects linear regression model. Some centres had complete three-point time trend data available for the periods of ISAAC Phase I to ISAAC Phase III to GAN Phase I, whereas others had only ISAAC Phase III to GAN Phase I, ISAAC Phase I to GAN Phase I, or ISAAC Phase I to ISAAC Phase III.

We included country-level and centre-level random intercepts in the mixed-effects linear regression models. The estimated time trend could therefore be interpreted as the within-centre absolute change in percentage point prevalence per decade. Data from both age groups were included in the one model to improve model efficiency. We included age group as an a-priori confounder and effect modifier to assess time trends in each age group separately.

We considered country income category and region as confounders and effect modifiers of the time trend. Therefore, our analyses controlled, by stratification, for age group, region of the world, and country income level. In all centres, boys and girls were approximately evenly distributed, so we did not control for sex. We also tested for evidence against a linear form for the time trend through the introduction of a quadratic term. The main models were additionally fitted to estimate the time trend in severe wheeze, exercise wheeze, night cough, and ever having asthma.

To consider the effect of the level of prevalence of current wheeze on the change in prevalence of current wheeze, we additionally fitted separate models that included level of prevalence in ISAAC Phase III (or midpoint if no ISAAC Phase III data were available).

### Role of the funding source

The funders of the study had no role in study design, data collection, data analysis, data interpretation, or writing of the report; and no role in the decision to submit the paper for publication.

## Results

Data were available for 27 centres, in 14 countries in all four regions (Africa and Eastern Mediterranean, America, Europe, and South-East Asia and Western Pacific) that had completed ISAAC Phase I, Phase III, or both and for which GAN Phase I methods and data checks were completed by Jan 29, 2021. Overall, 119 795 participants from GAN Phase I were included: 74 361 adolescents in 27 centres (response rate 90%, range 68–100) and 45 434 children in 19 centres (response rate 79%, range 55–100; table 1; appendixes 2, 3). For the 27 centres in GAN Phase I with data available for adolescents, 13 had data from ISAAC Phase III alone, 13 had data from both ISAAC Phases I and III, and one (Athens) had data for ISAAC Phase I alone. For the 19 centres in GAN Phase I with data available for children, nine had data from ISAAC Phase III alone, nine had data from both ISAAC Phases I and III, and one (Chandigarh) had data from ISAAC Phase I alone.

**Table 1:** Data collection details in each study centre

		**13–14-years age group**				**6–7-years age group**			
		Principal investigator	GAN response rate, %*	Mean data collection date*	Years between Phases†	Principal investigator	GAN response rate, %*	Mean data collection date*	Years between Phases†
Africa and Eastern Mediterranean									
	Nigeria (Ibadan)	A Falade	85·0%	May, 2018	16·7	..	..	..	..
	South Africa (Cape Town)	H J Zar	84·4%	August, 2017	15·2	..	..	..	..
	Sudan (Khartoum)	M Nour	99·9%	March, 2017	14·1	..	..	..	..
	Syria (Lattakia)	G Dib	99·6%	April, 2019	17·3	Y Mohammad	93·0%	May, 2019	16·2
	Region total	..	..	December, 2017	15·7	..	..	May, 2019	16·2
Americas									
	Chile (South Santiago)	J Mallol	81·9%	March, 2015	13·3	..	..	..	..
	Costa Rica (Costa Rica)	M Soto-Martinez	67·5%	February, 2018	16·1	M Soto-Martinez	64·5%	January, 2018	16·0
	Ecuador (Quito)	A Cabrera Aguilar	100·0%	April, 2019	15·9	..	..	..	..
	Mexico (Ciudad Victoria)	R García-Almaráz	82·3%	December, 2015	12·7	R García-Almaráz	81·5%	February, 2016	12·9
	Mexico (Mexicali)	J V Mérida-Palacio	83·7%	April, 2016	13·7	J V Mérida-Palacio	77·0%	March, 2016	13·3
	Mexico (Mexico City [North Area])	B E Del Río Navarro	93·8%	September, 2015	12·9	B E Del Río Navarro	86·7%	June, 2016	13·6
	Mexico (Monterrey)	S N González-Díaz	88·0%	December, 2017	16·7	..	..	..	..
	Mexico (Toluca Urban Area)	E M Navarrete-Rodriguez	98·1%	October, 2015	13·1	E M Navarrete-Rodriguez	95·7%	April, 2016	13·5
	Nicaragua (Managua)	J F Sánchez	90·5%	November, 2018	16·5	J F Sánchez	87·9%	November, 2018	16·4
	Region total	..	..	December, 2016	14·5	..	..	January, 2017	14·4
Europe									
	Spain (A Coruña)	A López-Silvarrey Varela	92·1%	January, 2019	15·2	A López-Silvarrey Varela	71·0%	January, 2019	15·3
	Spain (Bilbao)	C González Díaz	91·1%	September, 2018	16·8	C González Díaz	55·2%	August, 2018	16·7
	Spain (Cartagena)	L García-Marcos	73·8%	January, 2016	13·9	L García-Marcos	65·9%	January, 2016	14·0
	Region total	..	..	December, 2017	15·4	..	..	November, 2017	15·3
South-East Asia and Western Pacific									
	India (Bikaner)	M Sabir	90·1%	November, 2017	16·3	..	..	..	..
	India (Chandigarh)	M Singh	100·0%	October, 2017	15·9	..	..	..	..
	India (Jaipur)	V Singh	98·7%	November, 2017	16·3	V Singh	75·8%	November, 2017	16·3
	India (Kottayam)	T U Sukumaran	85·3%	October, 2017	15·3	T U Sukumaran	68·4%	December, 2017	15·5
	India (Lucknow)	S Awasthi	94·0%	October, 2017	16·0	S Awasthi	91·3%	October, 2017	15·9
	India (New Delhi)	S K Kabra	100·0%	November, 2017	16·0	S K Kabra	80·9%	January, 2018	16·1
	India (Pune)	S Salvi	99·6%	October, 2017	15·9	S Salvi	79·8%	October, 2017	15·9
	New Zealand (Auckland)	M I Asher	85·5%	October, 2018	16·7	M I Asher	63·7%	July, 2018	16·4
	Taiwan (Taipei)	J-L Huang	93·0%	October, 2017	15·8	J-L Huang	76·3%	October, 2017	15·8
	Thailand (Bangkok)	S Chinratanapisit	97·9%	September, 2017	16·1	S Chinratanapisit	86·3%	August, 2017	16·1
	Region total	..	..	November, 2017	16·0	..	..	November, 2017	16·0
World total		..	..	August, 2017	15·4	**..**	**..**	August, 2017	15·3

The Athens centre is not presented because its ISAAC data are from Phase I alone; the Chandigarh centre is not presented in the 6–7-years age group because its ISAAC data are from Phase I alone. For the 13–14-years age group, methodology notes for centres with GAN Phase I data alone are the following: no date of birth on the questionnaire (Ibadan, Taipei); response rate lower than 80% (Costa Rica, Cartagena); age and birth date showed high inconsistencies (Quito); fewer than ten schools used when ten or more schools were available (Ciudad Victoria, Mexico City [north area], Toluca Urban Area, Auckland, Bangkok); only age was reported reliably (Monterrey); single data entry (Monterrey); no age on the questionnaire (Chandigarh, New Delhi); questionnaires completed at home by parents (Kottayam); and more than 20% of questionnaires missing age and date of birth (Bangkok). For the 6–7-years age group, methodology notes for centres with GAN Phase I data alone are the following: response rate lower than 70% (Costa Rica, Bilbao, Cartagena, Kottayam, Auckland); no date of birth on the questionnaire (Taipei); no age on the questionnaire (New Delhi); fewer than ten schools used when ten or more schools were available (Bangkok); and more than 20% of questionnaires missing age and date of birth (Bangkok). GAN=Global Asthma Network. ISAAC=International Study of Asthma and Allergies in Childhood.

* GAN Phase I.

† ISAAC Phase III to GAN Phase I.

GAN Phase I was undertaken between March 2, 2015 and Feb 28, 2020, with the date of data collection varying by centre. There was an average of 15·4 years (range 12·7–17·3) between ISAAC Phase III and GAN Phase I and 22·7 years (19·5–25·5) between ISAAC Phase I and GAN Phase I; dates of data collection were similar between the adolescent group and children group.

For current wheeze, in GAN Phase I, the prevalence in adolescents ranged from 0·9% (New Delhi, India) to 21·3% (Cape Town, South Africa), with a mean of 10·4% (95% CI 7·8–12·8); and the prevalence in children ranged from 1·1% (Lucknow, India) to 23·2% (Costa Rica), with a mean of 9·9% (7·3–12·4; Table 2, Table 3). The Bland Altman plots showed that the underlying prevalence of current wheeze was not associated with the magnitude of change (appendix 1, p 6).

**Table 2:** 12-month prevalence of asthma symptoms and absolute change per decade for the 13–14-years age group in each study centre

		Number of individuals*	**Wheeze in past 12 months**			**Asthma ever**			**Severe asthma symptoms in past 12 months**			**Exercise wheeze in past 12 months**			**Night cough in past 12 months**		
			Prevalence, %*	Absolute change per decade, %†	SE change†	Prevalence, %*	Absolute change per decade, %†	SE change†	Prevalence, %*	Absolute change per decade, %†	SE change†	Prevalence, %*	Absolute change per decade, %†	SE change†	Prevalence, %*	Absolute change per decade, %†	SE change†
Africa and Eastern Mediterranean																	
	Nigeria (Ibadan)	2897	10·6%	−1·4%	−1·0	3·7%	−4·8%	−5·3	6·2%	−1·3%	−1·2	32·0%	−1·3%	−0·7	23·6%	−2·5%	−1·8
	South Africa (Cape Town)	3979	21·3%	0·6%	0·5	16·6%	1·4%	1·5	12·0%	1·7%	2·7	36·0%	2·2%	1·3	41·4%	3·1%	2·4
	Sudan (Khartoum)	1785	5·7%	−4·8%	−3·9	18·2%	1·9%	1·2	3·5%	−2·7%	−3·2	28·1%	9·6%	3·4	40·4%	14·4%	7·2
	Syria (Lattakia)	1215	19·8%	7·7%	4·4	10·9%	2·8%	4·9	10·6%	4·7%	4·3	35·8%	13·6%	4·6	54·4%	19·4%	8·6
	Region total	9876	15·1%	0·7%	NA	12·4%	0·1%	NA	8·6%	0·8%	NA	33·4%	5·4%	NA	37·6%	6·2%	NA
Americas																	
	Chile (South Santiago)	2750	13·4%	−2·7%	−3·2	13·7%	−1·7%	−2·4	3·9%	−1·4%	−3·2	16·7%	−3·0%	−3·5	32·9%	−5·7%	−5·6
	Costa Rica (Costa Rica)	1338	20·8%	−4·1%	−3·9	22·0%	−0·7%	−0·6	9·4%	−2·7%	−3·7	14·4%	−3·3%	−4·4	31·2%	3·0%	2·7
	Ecuador (Quito)	3000	6·3%	−7·2%	−6·5	4·5%	−1·5%	−2·5	3·0%	−1·4%	−2·5	18·2%	4·8%	3·3	28·0%	12·7%	9·4
	Mexico (Ciudad Victoria)	2468	13·3%	−0·9%	−0·4	8·6%	2·2%	2·6	5·8%	0·4%	0·7	16·6%	−3·9%	−1·5	25·8%	−5·6%	−2·0
	Mexico (Mexicali)	2479	14·7%	7·4%	11·5	8·7%	5·4%	11·6	7·5%	3·9%	8·4	21·3%	11·2%	7·3	25·7%	16·4%	11·7
	Mexico (Mexico City [North Area])	3375	8·9%	−0·8%	−0·6	7·4%	−0·4%	−0·4	3·7%	−0·2%	−0·3	15·5%	1·9%	1·3	14·8%	−13·2%	−4·7
	Mexico (Monterrey)	2641	12·5%	3·9%	6·9	11·3%	2·5%	4·2	5·5%	1·7%	4·0	23·1%	8·0%	7·6	31·2%	−2·6%	−1·1
	Mexico (Toluca Urban Area)	2650	5·7%	−0·7%	−0·7	6·2%	0·8%	1·8	2·3%	−0·9%	−1·3	11·0%	−5·2%	−1·6	16·1%	−3·4%	−1·2
	Nicaragua (Managua)	3131	16·9%	1·9%	2·3	20·1%	3·0%	4·1	9·8%	1·3%	2·2	21·9%	−2·6%	−2·6	43·8%	0·2%	0·2
	Region total	23 832	11·9%	−0·5%	NA	10·8%	0·9%	NA	5·4%	0·0%	NA	17·8%	1·2%	NA	27·5%	0·2%	NA
Europe																	
	Spain (A Coruña)	3462	16·5%	0·8%	1·1	20·6%	1·4%	1·9	8·1%	1·2%	2·5	21·3%	0·2%	0·2	35·7%	4·9%	4·6
	Spain (Bilbao)	3379	19·0%	3·1%	4·8	29·9%	4·8%	6·4	9·6%	2·2%	5·9	26·9%	2·8%	3·0	35·3%	8·9%	8·6
	Spain (Cartagena)	3437	10·2%	−0·3%	−0·5	14·9%	2·3%	3·4	4·1%	0·1%	0·3	13·9%	−0·8%	−0·9	23·6%	−2·8%	−3·0
	Region total	10 278	15·2%	1·4%	NA	21·7%	3·1%	NA	7·3%	1·3%	NA	20·7%	1·0%	NA	31·5%	4·0%	NA
South-East Asia and Western Pacific																	
	India (Bikaner)	2702	2·4%	−3·2%	−3·2	3·5%	−0·7%	−1·1	1·6%	−0·9%	−1·7	8·7%	−0·1%	−0·1	22·5%	−2·5%	−2·4
	India (Chandigarh)	3000	2·5%	−1·9%	−5·6	1·2%	−1·7%	−5·0	0·7%	−1·2%	−5·8	10·4%	3·2%	2·2	39·1%	7·8%	3·8
	India (Jaipur)	3060	6·8%	0·8%	1·1	6·2%	0·2%	0·3	2·1%	−0·5%	−1·6	9·8%	3·3%	5·9	45·8%	11·4%	8·2
	India (Kottayam)	2091	4·4%	−7·1%	−5·9	4·3%	−3·0%	−2·0	1·5%	−5·0%	−4·1	4·8%	−4·5%	−4·1	17·3%	−7·6%	−5·0
	India (Lucknow)	2969	1·6%	−2·6%	−4·6	1·3%	−1·2%	−2·6	0·8%	−1·2%	−3·1	9·5%	2·0%	1·5	22·7%	−4·9%	−1·5
	India (New Delhi)	3024	0·9%	−2·8%	−4·2	0·3%	−3·9%	−5·2	0·5%	−1·8%	−4·7	6·6%	1·2%	1·7	27·3%	13·1%	12·6
	India (Pune)	3030	4·6%	1·1%	2·2	7·9%	1·7%	2·3	2·0%	1·2%	5·1	10·6%	2·6%	2·7	33·0%	14·2%	9·0
	New Zealand (Auckland)	1885	14·9%	−4·6%	−3·4	22·6%	−3·2%	−3·0	5·1%	−2·2%	−3·6	22·7%	−5·8%	−5·2	24·9%	−3·6%	−2·6
	Taiwan (Taipei)	3474	9·2%	1·4%	2·9	14·2%	−1·7%	−2·7	3·3%	0·7%	2·7	25·0%	3·6%	4·8	27·7%	9·6%	13·5
	Thailand (Bangkok)	3206	12·5%	−0·9%	−0·7	8·8%	−4·4%	−5·9	5·8%	−0·4%	−0·6	14·8%	−2·0%	−1·4	30·0%	−0·7%	−0·2
	Region total	28 441	5·8%	−2·1%	NA	6·7%	−2·5%	NA	2·3%	−1·2%	NA	12·4%	−0·1%	NA	29·7%	4·3%	NA
	World total	72 427	10·4%	−0·7%	NA	11·0%	−0·3%	NA	4·9%	−0·2%	NA	18·2%	1·2%	NA	30·3%	3·2%	NA

The Athens centre is not presented because its ISAAC data are from Phase I alone. NA=not applicable. Methodology notes for centres with GAN Phase I data alone are the following: no date of birth on the questionnaire (Ibadan, Taipei); response rate lower than 80% (Costa Rica, Cartagena); age and birth date showed high inconsistencies (Quito); fewer than ten schools used when ten or more schools were available (Ciudad Victoria, Mexico City [north area], Toluca Urban Area, Auckland, Bangkok); only age was reported reliably (Monterrey); single data entry (Monterrey); no age on the questionnaire (Chandigarh, New Delhi); questionnaires completed at home by parents (Kottayam); and more than 20% of questionnaires missing age and date of birth (Bangkok). GAN=Global Asthma Network. ISAAC=International Study of Asthma and Allergies in Childhood.

* GAN Phase I.

† ISAAC Phase III to GAN Phase I.

**Table 3:** 12-month prevalence of asthma symptoms and absolute change per decade for the 6–7-years age group in each study centre

			**Wheeze in past 12 months**			**Asthma ever**			**Severe asthma symptoms in past 12 months**			**Exercise wheeze in past 12 months**			**Night cough in past 12 months**		
		Number of individuals*	Prevalence, %*	Absolute change per decade, %†	SE change†	Prevalence, %*	Absolute change per decade, %†	SE change†	Prevalence, %*	Absolute change per decade, %†	SE change†	Prevalence, %*	Absolute change per decade, %†	SE change†	Prevalence, %*	Absolute change per decade, %†	SE change†
Africa and Eastern Mediterranean																	
	Syria (Lattakia)	1116	10·8%	3·8%	5·1	11·9%	4·9%	5·2	5·4%	1·7%	3·2	11·6%	5·5%	6·7	29·1%	8·6%	4·4%
	Region total	1116	10·8%	3·8%	NA	11·9%	4·9%	NA	5·4%	1·7%	NA	11·6%	5·5%	NA	29·1%	8·6%	NA
Americas																	
	Costa Rica (Costa Rica)	1936	23·2%	−9·0%	−7·5	29·3%	0·9%	0·6	13·2%	−3·2%	−4·1	12·2%	−3·0%	−3·5	45·9%	5·6%	5·5%
	Mexico (Ciudad Victoria)	2444	11·7%	2·4%	3·9	6·5%	1·4%	2·5	5·8%	1·8%	4·4	10·0%	4·5%	7·1	22·7%	−4·6%	−4·3%
	Mexico (Mexicali)	2001	14·0%	3·9%	5·7	7·5%	−0·2%	−0·4	7·6%	3·3%	5·9	14·8%	7·0%	10·3	23·0%	−3·4%	−2·8%
	Mexico (Mexico City [North Area])	2515	10·6%	2·8%	4·2	5·1%	0·5%	1·0	4·3%	1·5%	4·1	9·1%	4·2%	7·5	19·8%	−7·9%	−5·6%
	Mexico (Toluca Urban Area)	2712	6·4%	0·4%	0·4	3·4%	1·0%	2·6	2·9%	0·7%	1·4	6·5%	2·2%	3·2	18·0%	−0·6%	−0·5%
	Nicaragua (Managua)	3162	12·2%	−3·0%	−3·0	14·0%	−1·8%	−1·9	5·9%	−2·1%	−3·2	7·9%	−4·9%	−5·9	32·5%	−7·2%	−3·7%
	Region total	14 770	12·5%	−1·5%	NA	10·4%	−0·4%	NA	6·3%	−0·3%	NA	9·7%	0·8%	NA	26·5%	−3·4%	NA
Europe																	
	Spain (A Coruña)	3407	11·0%	−1·3%	−2·3	9·7%	−2·6%	−4·5	4·4%	−0·2%	−0·4	4·9%	−0·8%	−2·0	30·9%	4·8%	5·1%
	Spain (Bilbao)	2707	10·9%	−0·9%	−1·4	22·7%	1·2%	1·4	4·1%	0·2%	0·4	6·4%	−0·1%	−0·3	27·8%	4·2%	5·0%
	Spain (Cartagena)	3509	11·7%	0·5%	0·9	10·3%	−0·3%	−0·6	4·4%	0·2%	0·4	6·1%	0·7%	1·7	27·4%	4·7%	5·6%
	Region total	9623	11·2%	−0·6%	NA	13·6%	−1·1%	NA	4·3%	0·1%	NA	5·7%	−0·1%	NA	28·7%	4·6%	NA
South-East Asia and Western Pacific																	
	India (Jaipur)	2296	2·4%	−2·0%	−2·1	2·2%	−2·2%	−2·3	0·8%	−1·7%	−2·0	2·5%	−0·6%	−0·5	19·9%	−2·9%	−2·3%
	India (Kottayam)	2099	5·5%	−11·3%	−13·4	3·5%	−5·2%	−3·7	2·9%	−5·3%	−4·8	1·8%	−7·1%	−6·2	20·9%	−0·3%	−0·2%
	India (Lucknow)	2969	1·1%	−1·5%	−3·3	0·6%	−1·1%	−3·7	0·5%	−0·6%	−2·7	1·9%	0·2%	0·6	6·7%	−5·7%	−5·0%
	India (New Delhi)	2516	3·5%	−1·6%	−2·6	0·4%	−3·9%	−5·7	0·8%	−1·1%	−5·8	2·7%	−0·2%	−0·6	22·9%	9·8%	7·8%
	India (Pune)	2404	2·1%	−1·2%	−3·4	1·8%	−1·0%	−3·6	0·7%	0·4%	4·1	4·4%	0·8%	1·7	19·8%	5·0%	5·3%
	New Zealand (Auckland)	1538	17·4%	−3·1%	−3·6	19·2%	−5·5%	−4·5	6·7%	−2·0%	−3·4	12·0%	−2·1%	−2·3	24·8%	−2·7%	−2·7%
	Taiwan (Taipei)	3036	13·6%	2·4%	4·2	14·5%	0·1%	0·2	3·7%	1·0%	3·7	6·9%	1·3%	3·8	32·8%	7·4%	14·7%
	Thailand (Bangkok)	3067	14·6%	−0·2%	−0·3	6·1%	−2·9%	−4·2	6·8%	0·9%	1·9	3·0%	−1·7%	−3·6	24·2%	−4·2%	−5·1%
	Region total	19 925	7·4%	−2·5%	NA	5·6%	−3·4%	NA	2·8%	−1·0%	NA	4·1%	−1·3%	NA	21·4%	0·5%	NA
World total		45 434	9·9%	−1·5%	NA	9·0%	−1·6%	NA	4·3%	−0·5%	NA	6·4%	−0·2%	NA	24·8%	0·5%	NA

The Chandigarh centre is not presented because its ISAAC data are from Phase I alone. NA=not applicable. Methodology notes for centres with GAN Phase I data alone are the following: response rate lower than 70% (Costa Rica, Bilbao, Cartagena, Kottayam, Auckland); no date of birth on the questionnaire (Taipei); no age on the questionnaire (New Delhi); fewer than ten schools used when ten or more schools were available (Bangkok); and more than 20% of questionnaires missing age and date of birth (Bangkok). GAN=Global Asthma Network. ISAAC=International Study of Asthma and Allergies in Childhood.

* GAN Phase I.

† ISAAC Phase III to GAN Phase I.

We assessed the changes within centres in absolute prevalence of current wheeze from ISAAC (Phases I and III) to GAN Phase I (figure). Within each age group, most centres in both age groups showed significant (≥2 SE up or down) changes in current wheeze and severe asthma symptoms. For prevalence of current wheeze in adolescents, ten centres showed an SE decrease of 2 or more, seven showed an SE increase of 2 or more, and nine showed an SE change lower than 2 (appendix 1, p 8). For prevalence of current wheeze in children, nine centres showed an SE decrease of 2 or more, five showed an SE increase of 2 or more, and four showed an SE change lower than 2 (appendix 1, p 12). Within centres and in both age groups, the pattern of changes in prevalence of severe asthma symptoms (appendix 1, pp 9, 13) and ever having asthma (appendix 1, pp 10, 14) was similar to the changes in current wheeze. Additionally, the pattern of changes in prevalence of exercise wheeze and night cough was similar to those in current wheeze in both age groups (Table 2, Table 3).

**Figure:**
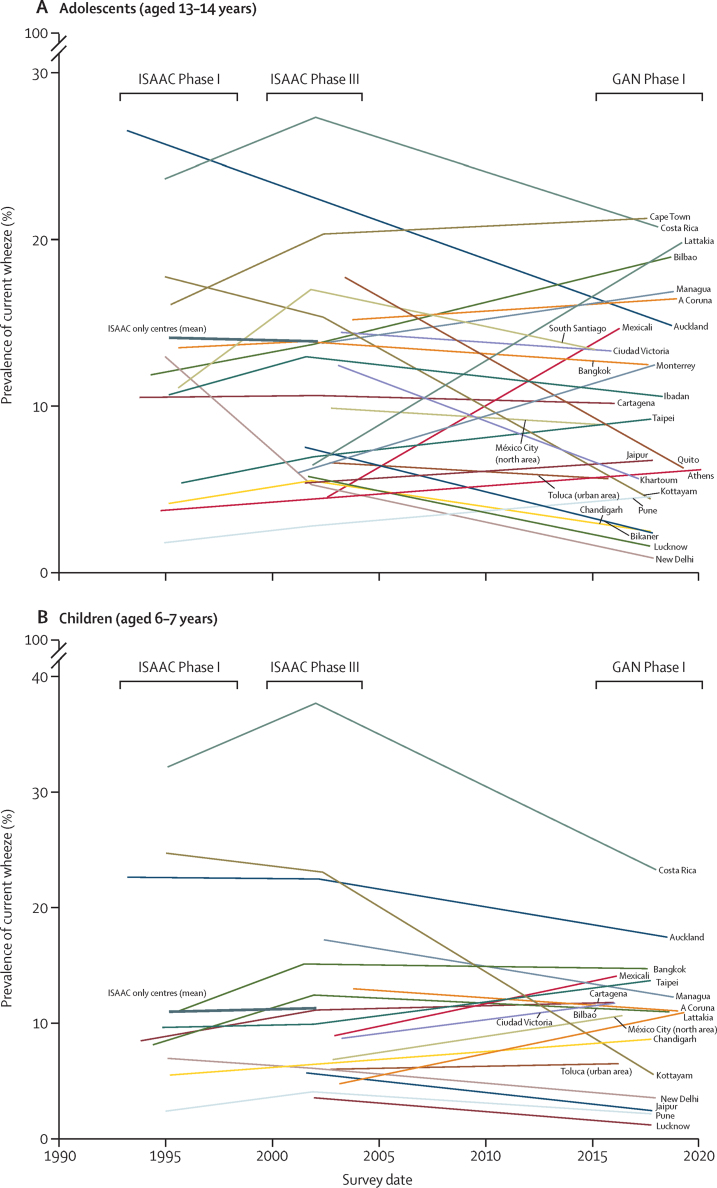
Absolute changes over time in prevalence of current wheeze for adolescents (A) and children (B) by survey date Each thin line represents one centre. The thick line shows the average absolute change from ISAAC Phase I to Phase III for those centres that did not participate in GAN Phase I. The span of the years of data collection for ISAAC Phase I, ISAAC Phase III, and GAN Phase I are shown. GAN=Global Asthma Network. ISAAC=International study of Asthma and Allergies in Childhood.

The regression models examined whether within-centre changes were, collectively, compatible with chance or real trends. The models showed the effects of age group, region, and country income group upon time trends in current wheeze to be minimally altered by adjustment for their mutual confounding (data not shown). Although we observed no evidence for age group as an effect modifier (p=0·67), we decided a priori to stratify on this variable due to the importance of showing age group-specific results. We observed evidence of an additional effect modification with both income group (p=0·002) and region (p=0·0002), but the low number of centres in each stratum meant that we did not consider these effect modifiers together.

After stratifying by age group, adolescents showed a decrease in percentage point prevalence per decade of severe asthma symptoms and an increase in ever having asthma and night cough, which was also found in children (table 4). Stratification by country income group (adjusted for region) showed a decrease in prevalence of current wheeze among low-income countries in both age groups. However, we found an increase in prevalence of current wheeze among both age groups in lower-middle-income countries, but no change in upper-middle-income and high-income countries (table 4). Stratification by world region (adjusted for income group) showed an increase in prevalence of current wheeze in the Africa and Eastern Mediterranean region for both age groups, whereas the South-East Asia and Western Pacific regions showed decreased prevalence of current wheeze (table 4). We observed no evidence of a change in prevalence for the regions of America and Europe in either age group. We assessed nine strata across two age groups and five symptom outcomes, but not all of these are statistically independent. Within-centre trends in prevalence of related symptoms, and of a given symptom across age groups, might be correlated.

**Table 4:** Model results of estimated change in all asthma related outcomes over 10 years

		**Outcome**					Number of surveys*in strata
		Current wheeze	Severe asthma symptoms	Exercise wheeze	Night cough	Asthma ever	
**Model stratified by age group**							
Age 6–7 years		−0·22 (−1·00 to 0·57)	−0·24 (−0·63 to 0·15)	0·78 (−0·31 to 1·87)	3·21 (1·80 to 4·62)	0·56 (−0·13 to 1·24)	161
Age 13–14 years		−0·43 (−1·10 to 0·23)	−0·37 (−0·69 to −0·04)	0·34 (−0·58 to 1·26)	4·25 (3·06 to 5·44)	1·25 (0·67 to 1·83)	255
**Model stratified by age group and income group**							
Age 6–7 years							
	Low income	−1·37 (−2·47 to −0·27)	−0·84 (−1·37 to −0·30)	−0·02 (−1·54 to 1·50)	3·33 (1·34 to 5·32)	−1·56 (−2·48 to −0·65)	31
	Lower-middle income	1·99 (0·33 to 3·66)	1·32 (0·51 to 2·12)	3·66 (1·36 to 5·96)	5·29 (2·28 to 8·29)	0·06 (−1·33 to 1·44)	15
	Upper-middle income	0·50 (−0·82 to 1·82)	0·20 (−0·44 to 0·84)	1·66 (−0·17 to 3·48)	1·41 (−0·98 to 3·80)	1·19 (0·09 to 2·28)	43
	High income	−0·22 (−1·24 to 0·80)	−0·39 (−0·88 to 0·11)	0·25 (−1·16 to 1·66)	3·48 (1·63 to 5·32)	2·07 (1·22 to 2·92)	72
Age 13–14 years							
	Low income	−1·67 (−2·70 to −0·64)	−1·03 (−1·53 to −0·53)	−0·58 (−2·00 to 0·85)	4·31 (2·45 to 6·17)	−0·84 (−1·70 to 0·02)	47
	Lower-middle income	1·69 (0·13 to 3·25)	1·13 (0·37 to 1·88)	3·10 (0·95 to 5·25)	6·26 (3·46 to 9·07)	0·78 (−0·52 to 2·08)	40
	Upper-middle income	0·19 (−1·06 to 1·45)	0·01 (−0·60 to 0·62)	1·10 (−0·63 to 2·83)	2·38 (0·12 to 4·65)	1·91 (0·86 to 2·95)	62
	High income	−0·52 (−1·47 to 0·43)	−0·58 (−1·04 to −0·12)	−0·31 (−1·62 to 1·00)	4·45 (2·73 to 6·17)	2·79 (2·00 to 3·58)	106
**Model stratified by age group and grouped region**							
Age 6–7 years							
	Africa and Eastern Mediterranean	2·61 (0·76 to 4·46)	1·46 (0·57 to 2·35)	4·99 (2·41 to 7·57)	6·64 (3·28 to 9·99)	0·21 (−1·38 to 1·79)	10
	America	0·01 (−1·29 to 1·31)	0·13 (−0·50 to 0·76)	1·23 (−0·56 to 3·03)	1·43 (−0·92 to 3·77)	0·88 (−0·24 to 1·99)	29
	Europe†	1·08 (−0·08 to 2·24)	0·43 (−0·13 to 0·99)	1·60 (−0·01 to 3·21)	4·67 (2·57 to 6·78)	2·73 (1·74 to 3·73)	64
	South-East Asia and Western Pacific	−1·35 (−2·28 to −0·41)	−0·96 (−1·41 to −0·51)	−0·33 (−1·64 to 0·98)	2·71 (1·01 to 4·42)	−0·69 (−1·49 to 0·11)	58
Age 13–14 years							
	Africa and Eastern Mediterranean	2·09 (0·40 to 3·78)	1·15 (0·33 to 1·96)	4·15 (1·80 to 6·51)	7·41 (4·34 to 10·47)	0·78 (−0·68 to 2·23)	34
	America	−0·51 (−1·73 to 0·71)	−0·19 (−0·77 to 0·40)	0·39 (−1·29 to 2·07)	2·20 (0·00 to 4·40)	1·45 (0·40 to 2·50)	50
	Europe†	0·56 (−0·51 to 1·63)	0·11 (−0·40 to 0·63)	0·76 (−0·73 to 2·25)	5·45 (3·50 to 7·39)	3·30 (2·38 to 4·22)	100
	South-East Asia and Western Pacific	−1·87 (−2·78 to −0·96)	−1·28 (−1·72 to −0·84)	−1·17 (−2·44 to 0·10)	3·49 (1·83 to 5·14)	−0·12 (−0·90 to 0·66)	71

Data are absolute percentage points difference (95% CI). Results from mixed model with random intercepts for country and centre, incorporating effect modification, fully adjusted for age group, income group, and grouped region (n=416). ISAAC=International Study of Asthma and Allergies in Childhood.

* N=number of surveys contributing to each stratified analysis.

† Malta was in ISAAC's Eastern Mediterranean region but has been moved to the European region in modelling for this analysis.

The results of the models for the other four outcome variables showed that the pattern for prevalence of severe asthma symptoms and exercise wheeze was similar to that of current wheeze (table 4). However, ever having asthma showed a different pattern, with increases in high-income countries and Europe. For night cough, we found a consistent pattern of increase in point prevalence in both age groups, all country income groups, and most regions.

## Discussion

To our knowledge, GAN Phase I has provided the first standardised global estimates of trends over time in prevalence and severity of asthma symptoms in school-aged children since 2003. The study included almost 120 000 children from 27 centres in 14 countries using the same instruments and methods as ISAAC.^9^, ^10^ We identified a substantial burden of asthma symptoms in school-aged children: in both age groups, about one in ten had wheeze in the preceding year, of whom almost half had severe symptoms. We modelled all ISAAC Phases I and III and GAN Phase I prevalence data over time to determine patterns of change over nearly three decades (1993–2020) and found prevalence and severity to vary by age group, country income, region, and centre.

We observed change in prevalence of all asthma symptoms by 2 SE or more in most centres, suggesting more than just random variation. In seven countries, we found a decrease in prevalence of most asthma symptoms, whereas increases were found in seven countries. There is evidence from the 27-year modelling of an overall decrease in prevalence for severe asthma symptoms in adolescents and an overall increased report of ever having asthma and night cough in both age groups. We also found regional differences, with evidence of increasing prevalence of current wheeze and severe asthma symptoms in Africa and Eastern Mediterranean and decreasing mean prevalence in South-East Asia and Western Pacific regions. Country income was associated with changes in prevalence of asthma symptoms, with low-income countries showing a significant decrease but lower-middle-income countries showing an increase, with no evidence of change in more affluent countries. These broad patterns overlie a substantial heterogeneity of trends both between and within countries. This limits the generalisability of conclusions from any single centre or country.

The interpretation of these patterns is complex because a wide range of risk factors exists and environmental changes are at play. Some centres had changes of particular interest. For example, large variations were found within India, and the reasons for this are subject to a national study. A few centres with high prevalence saw a progressive decrease from ISAAC Phases I to III^7^, ^8^ to GAN Phase I. Some low-prevalence centres showed increased prevalence of asthma symptoms, including severe asthma symptoms, contributing to the burden of asthma. A large increase in prevalence was found in Lattakia, in war-torn Syria. In two centres (Cape Town and Bilbao), the increase in prevalence of severe asthma symptoms was more than that of asthma symptoms; whether this is due to barriers to asthma care remains to be investigated. The influence of asthma programmes, accessibility of asthma medicines, or other environmental changes^1^ on these variations needs to be determined. National asthma programmes can be effective ways of reducing the burden of severe asthma symptoms: Costa Rica, India, and Taiwan reported national asthma strategies in 2013–14,^22^ which might be a factor in the decreased burden in Costa Rica and India, but not in Taiwan. The role of macro-environmental changes affecting populations is worthy of exploration.

The Global Burden of Disease (GBD) Study has reported estimates of global asthma prevalence (for all ages) over a similar period as this analysis. The GBD case definition for asthma differed from ours in that it was a reported diagnosis by a physician (which varies between countries) combined with wheezing in the preceding 12 months. The GBD estimates over 2000–17 showed large and unexplained variations between 220·4 and 339·4 million people.^2^, ^23^, ^24^, ^25^, ^26^, ^27^, ^28^ Since 2003, no new worldwide standardised studies of asthma symptom prevalence were done until GAN Phase I, which also included additional 36 centres in 11 countries that did not undertake ISAAC, not included in this Article, and should contribute to future GBD analyses.

Our study has many strengths, especially the standardised methods used to estimate asthma prevalence from symptoms in a wide range of settings in the world.^6^ Tight quality control checks along with the same key personnel in ISAAC and GAN meant that replication of the standardised methods from ISAAC Phase I to Phase III to GAN Phase I was successful, as evidenced by the low number of centres excluded or with footnotes related to the methods. Response rates were high. The study includes many centres from low-income, middle-income, and high-income countries from all regions of the world except North America. In about half of the centres, GAN Phase I was the first paired standardised study of asthma symptom prevalence; these included centres in five countries with no previous time-trend data (centres in Ecuador, Greece, Nicaragua, Sudan, and Syria), and nine centres that had no previous time-trend data (although different centres in those countries had provided ISAAC time-trend data).^8^

Our study has some limitations, which include the smaller number of GAN Phase I centres compared with that of ISAAC Phase I to Phase III, which makes it difficult to generalise the findings to global prevalence changes. Centres were self-selecting, and thus not representative of countries except for Costa Rica, which was a whole country study. Within an individual country, wide differences of prevalence can occur such as rural versus urban locations and high-income areas in a low-income or middle-income country. Despite the simplicity of the GAN and ISAAC approach, these surveys are more difficult to undertake now than at the time of ISAAC Phases I and III. Factors that increase this difficulty include reluctance of schools to being involved in research due to increased curriculum demands; more stringent ethical requirements, meaning that obtaining passive consent for adolescents is less easy than in previous decades;^16^ funders not prioritising population-based research; parents being busier and having less time for participation; and epidemics or pandemics (currently COVID-19) and conflicts that disrupt schools and people's lives.

Future GAN Phase I reports will contribute an estimate of the global burden of asthma, which will include the GAN Phase I centres in this Article as well as centres without time-trend data, and analyses in children and adolescents assessing rhinoconjunctivitis and eczema and other asthma risk factors, similar to ISAAC Phase III reports, as well as studies in adults. Extensive risk factor analyses were undertaken in ISAAC Phase III.^29^, ^30^ Equivalent analyses will be undertaken with use of GAN Phase I data in conjunction with ISAAC Phase III data to determine to what extent changes in risk factor prevalences over time can account for the observed changes in prevalence and severity of asthma symptoms. We will also examine the relationship of management of asthma and symptoms. Abundant evidence exists that in many locations in the world, asthma management is suboptimal, contributing to a relatively high prevalence of severe asthma symptoms,^1^, ^3^ and there are strategies to improve this even in localities within low-resource settings.^1^, ^31^ As the legacy of severe asthma in childhood can be chronic obstructive pulmonary disease in adults, reducing the burden of severe asthma symptoms in children is of crucial importance.^2^

These data suggest that, while the overall worldwide prevalence of asthma symptoms is relatively stable, about one in 20 school-aged children have severe asthma symptoms, and they need to gain better asthma control to lessen the associated avoidable asthma morbidity and mortality; little has changed over 27 years. The UN 2030 Sustainable Development Goal 3 aims to “ensure healthy lives and promote wellbeing at all ages”.^1^ Our findings emphasise the urgency of ensuring that the high worldwide burden of severe asthma symptoms in children is mitigated by enabling equitable and affordable access to the effective therapies for asthma that have been available to those who can afford them for decades.

## Data sharing

ISAAC data are already deposited for wider use. The study protocol, including a recommended informed consent form and statistical analysis plan, are in the public domain (https://beta.ukdataservice.ac.uk/datacatalogue/studies/study?id=8131). The GAN Phase I data, including de-identified individual participant data, will be made available on the GAN website within 12 months of the publication of all GAN Phase I analyses. Access will require a formal request through the website, a written proposal, and a signed data access agreement.

For the **GAN website** see http://www.globalasthmanetwork.org/

## Declaration of interests

KM reports receiving advisory board fees from AstraZeneca, outside the submitted work. GBM reports grants and non-financial support from AstraZeneca and grants from GlaxoSmithKline Australia and Novartis Australia, outside the submitted work. All other authors declare no competing interests.
