
JOURNAL INFORMATION
==============================
NLM Title Abbreviation: Lancet
Journal ID: Lancet
NLM Journal ID: 2985213R

Lancet (London, England)

ISSN: 0140-6736
EISSN: 1474-547X

ARTICLE INFORMATION
==============================
PMCID: PMC8593551
DOI: 10.1016/S0140-6736(21)02389-8
Article ID: S0140-6736(21)02389-8
Article version: 1
Subjects: Department of Error

Department of Error

Print publication date: 2021 Nov 6
Volume: 398
Issue: 10312
First page: 1686
Last page: 1686
Copyright: © 2021 The Author(s). Published by Elsevier Ltd.
Copyright year: 2021
Copyright holder: Elsevier Ltd
License: This is an open access article under the CC BY license (http://creativecommons.org/licenses/by/4.0/).
License URL: https://creativecommons.org/licenses/by/4.0/
Erratum for: Worldwide trends in the burden of asthma symptoms in school-aged children: Global Asthma Network Phase I cross-sectional study 398 10311 30 10 2021 1569 1580 Lancet (London, England) 10.1016/S0140-6736(21)01450-1 PMC8573635 34755626

==============================
Asher MI, Rutter CE, Bissell K, et al. Worldwide trends in the burden of asthma symptoms in school-aged children: Global Asthma Network Phase I cross-sectional study. Lancet 2021; 398: 1569–80—In this Article, the spelling of author Karen Bissell's name was incorrect. This correction has been made to the online version as of Nov 4, 2021.

