# Supplementary material for: Worldwide trends in the burden of asthma symptoms in school-aged children: Global Asthma Network Phase I cross-sectional study
Source: Lancet. 2021 Oct 30;398(10311):1569–80. doi: 10.1016/S0140-6736(21)01450-1 (PMC8573635; doi:10.1016/S0140-6736(21)01450-1)
Supplement: Supplementary appendix [file mmc1.pdf]

THE LANCET

Supplementary appendix

This appendix formed part of the original submission and has been peer reviewed. We post it as supplied by the authors.

Supplement to: Asher MI, Rutter CE, Bissell K, et al. Worldwide trends in the burden of asthma symptoms in school-aged children: Global Asthma Network Phase I cross-sectional study. *Lancet* 2021; **398:** 1569–80.

Global Asthma Network Phase I Study Group:

Global Asthma Network Steering Group: MI Asher, Department of Paediatrics: Child and Youth Health, Faculty of Medical and Health Sciences, University of Auckland, Private Bag 92019, Auckland, New Zealand; K Bissell, School of Population Health, Faculty of Medical and Health Sciences, University of Auckland, Auckland, New Zealand; C-Y Chiang, International Union Against Tuberculosis and Lung Disease, Paris, France; and Division of Pulmonary Medicine, Department of Internal Medicine, Wan Fang Hospital, Taipei Medical University; and Division of Pulmonary Medicine, Department of Internal Medicine, School of Medicine, College of Medicine, Taipei Medical University111 Hsin-Long Road, Section 3, Taipei, 116, Taiwan; A El Sony, Epidemiological Laboratory for Public Health and Research, Khartoum 3 Block3-Building 11, Khartoum, Sudan; E Ellwood, Department of Paediatrics: Child and Youth Health, Faculty of Medical and Health Sciences, Private Bag 92019, University of Auckland, Auckland, New Zealand; P Ellwood, Department of Paediatrics: Child and Youth Health, Faculty of Medical and Health Sciences, Private Bag 92019, University of Auckland, Auckland, New Zealand; L García-Marcos, Pediatric Allergy and Pulmonology Units, Virgen de la Arrixaca University Children‘s Hospital, University of Murcia and IMIB Bioresearch Institute, Murcia; and ARADyAL Allergy Network, Edificio Departamental-Laib, Avenida Buenavista s/n, 30120 El Palmar, 30394 Murcia Spain; GB Marks, Respiratory & Environmental Epidemiology, University of New South Wales, Goulburn St, Sydney 2085, Sydney, Australia; R Masekela, Department of Paediatrics and Child Health, University of Kwazulu-Natal, Pretoria, South Africa; E Morales, Department of Public Health Sciences, University of Murcia, and IMIB Bio-health Research Institute, Murcia, Edificio Departamental-Laib, Avenida Buenavista s/n, 30120 El Palmar, 30394 Murcia, Spain; K Mortimer, Liverpool School of Tropical Medicine, Pembroke Place, Liverpool L3 5QA, United Kingdom; N Pearce, Department of Medical Statistics, London School of Hygiene & Tropical Medicine, Keppel Street, London WC1E 7HT, United Kingdom; DP Strachan, Population Health Research Institute, St George’s, University of London, Cranmer Terrace, London SW17 0RE, United Kingdom.

Global Asthma Network International Data Centres: GAN Global Centre: P Ellwood, E Ellwood, MI Asher, Department of Paediatrics: Child and Youth Health,

Faculty of Medical and Health Sciences, Private Bag 92019, University of Auckland, Auckland, New Zealand; **Murcia, Spain:** L García-Marcos, Pediatric Allergy and Pulmonology Units, Virgen de la Arrixaca University Children‘s Hospital, University of Murcia and IMIB Bioresearch Institute, Murcia; and ARADyAL Allergy Network, Edificio Departamental-Laib, Murcia, Spain; V Pérez-Fernández, Department of Paediatrics, University of Murcia; and IMIB Bio-health Research Institute, Murcia, Edificio Departamental-Laib, Avenida Buenavista s/n, 30120 El Palmar, 30394 Murcia Spain; E Morales, Department of Public Health Sciences, University of Murcia, and IMIB Bio-health Research Institute, Murcia, Edificio Departamental-Laib, Avenida Buenavista s/n, 30120 El Palmar, 30394 Murcia, Spain; A Martinez-Torres, Paediatric Allergy and Pulmonology Units and Nurse Research Group, Virgen de la Arrixaca University Children‘s Hospital, University of Murcia and IMIB Bio-health Research Institute, Murcia, Edificio Departamental-Laib, Avenida Buenavista s/n, 30120 El Palmar, 30394 Murcia, Spain; **London, United Kingdom:** DP Strachan, Population Health Research Institute, St George’s, University of London, Cranmer Terrace, London SW17 0RE, United Kingdom; N Pearce, S Robertson, CE Rutter, Department of Medical Statistics, London School of Hygiene & Tropical Medicine, Keppel Street, London WC1E 7HT, United Kingdom; RJ Silverwood, Department of Medical Statistics, London School of Hygiene & Tropical Medicine, Keppel Street, London WC1E 7HT, United Kingdom and Centre for Longitudinal Studies, UCL Social Research Institute, University College London, 20 Bedford Way, London WC1H 0AL, United Kingdom.

Global Asthma Network Principal Investigators: Chile: J Mallol, University of Santiago de Chile (USACH), Santiago, (South Santiago); Costa Rica: M Soto-Martinez, University of Costa Rica (Costa Rica); Ecuador: A Cabrera Aguilar Respiraclinic, Planta Baja, Código (Quito); Greece: K Douros, National and Kapodistrian University of Athens (Athens); India: M Sabir, Kothari Medical & Research Institute (Bikaner); M Singh, Postgraduate Institute of Medical Education and Research (Chandigarh); V Singh*, Asthma Bhawan (Jaipur); TU Sukumaran, Pushpagiri Institute Of Medical Sciences And Research, Thiruvalla (Kottayam); S Awasthi, King George's Medical University (Lucknow); SK Kabra, All India Institute of

Medical Sciences (New Delhi); S Salvi, Chest Research Foundation (Pune); Mexico: R García-Almaráz, Hospital Infantil de Tamaulipas (Ciudad Victoria); JV Mérida-Palacio, Centro de Investigacion de Enfermedades Alergicas y Respiratorias (Mexicali); BE Del Río Navarro*, Service of Allergy and Clinical immunology, Hospital Infantil de México (Mexico City North); SN González-Díaz, Centro Regional de Alergia e Immunología Clínica, Hospital Universitario “Dr. José Eleuterio González”, Universidad Autónoma de Nuevo León (Monterrey); EM Navarrete-Rodriguez, Hospital Infantil de Mexico Federico Gomez (Toluca urban); New Zealand: MI Asher, Department of Paediatrics: Child and Youth Health, Faculty of Medical and Health Sciences, University of Auckland (Auckland); Nicaragua: JF Sánchez, Hospital Infantil Manuel de Jesús Rivera (Managua); Nigeria: A Falade, University of Ibadan and University College Hospital (Ibadan); South Africa: HJ Zar, SA MRC Unit on Child & Adolescent Health (Cape Town); Spain: A López-Silvarrey Varela, Fundacion Maria Jose Jove (A Coruña); C González Díaz, Departament of Paediatrics, Universidad del País Vasco UPV /EHU, Bilbao, Spain (Bilbao); L García-Marcos*, Pediatric Allergy and Pulmonology Units, Virgen de la Arrixaca University Children‘s Hospital, University of Murcia and IMIB Bioresearch Institute, Murcia; and ARADyAL Allergy Network, Edificio Departamental-Laib, Murcia, Spain (Cartagena); Sudan: M Nour, Epidemiological Laboratory (Epi-Lab) for Public Health, Research and Development, Khartoum, Sudan (Khartoum); Syrian Arab Republic: G Dib, Lattakia University (Lattakia 13-14); Y Mohammad*, National Center for research and training for chronic respiratory disease and co_morbidities (Lattakia 6-7); Taiwan: J-L Huang, Department of Pediatrics, Chang Gung Memorial Hospital, New Taipei Municipal TuChen Hospital, and Chang Gung University (Taipei); Thailand: S Chinratanapisit, Department of Pediatrics, Bhumibol Adulyadej Hospital, Royal Thai Air Force, Bangkok, Thailand (Bangkok).

* National Coordinators

Global Asthma Network National Co-ordinators not named above

Costa Rica: ME Soto-Quirós, University of Costa Rica, Costa Rica; Sudan: A El-Sony, Epidemiological Laboratory for Public Health and Research, Khartoum 3 Block3-Building 11, Khartoum, Sudan; Thailand: P Vichyanond, Mahidol University, Bangkok, Thailand.

ISAAC Phase Three Principal Investigators: Chile: P Aguilar, Hospital CRS El Pino, San Bernardo, Santiago, Chile (South Santiago); Costa Rica: ME Soto-Quirós*, University of Costa Rica (Costa Rica); Ecuador: S Barba* AXXIS-Medical Centre SEAICA (Quito); India: M Sabir, Kothari Medical & Research Institute (Bikaner); L Kumar†, Department of Pediatrics (Chandigarh); V Singh, Asthma Bhawan (Jaipur); TU Sukumaran, PIMS Thiruvalla (Kottayam); S Awasthi, King George's Medical University (Lucknow); SK Sharma, All India Institute of Medical Sciences (New Delhi [7]); NM Hanumante, Bharati Vidyapeeth Medical College (Pune); Mexico: R García-Almaráz, Hospital Infantil de Tamaulipas (Ciudad Victoria); JV Merida-Palacio, Centro de Investigacion de Enfermedades Alergicas y Respiratorias (Mexicali Valley); BE Del-Río-Navarro, Service of Allergy and Clinical immunology, Hospital Infantil de México (Ciudad de México [1]); SN González-Díaz, Centro Regional de Alergia e Immunología Clínica, Hospital Universitario “Dr. José Eleuterio González”, Universidad Autónoma de Nuevo León (Monterrey); FJ Linares-Zapién, Centro De Enfermedades Alergicas Y Asma de Toluca (Toluca); New Zealand: MI Asher*, Department of Paediatrics: Child and Youth Health, University of Auckland (Auckland); Nicaragua: JF Sánchez*, Hospital Infantil Manuel de Jesús Rivera (Managua); Nigeria: BO Onadeko, (Ibadan); South Africa: HJ Zar*, University of Cape Town (Cape Town); Spain: A López-Silvarrey Varela, Fundacion Maria Jose Jove (A Coruña); C González Díaz, Departament de Paediatrics, Universidad del País Vasco UPV /EHU, Bilbao, Spain (Bilbao); L García-Marcos*, Pediatric Allergy and Pulmonology Units, Virgen de la Arrixaca University Children‘s Hospital, University of Murcia and IMIB Bioresearch Institute, Murcia; and ARADyAL Allergy Network, Edificio Departamental-Laib, Murcia, Spain (Cartagena); Sudan: OAA Musa, Faculty of Medicine, National Ribat University, Khartoum, Sudan (Khartoum); Syrian Arab Republic: Y Mohammad, National Center for Research and Training in Chronic Respiratory Diseases - Tishreen University (Lattakia); Taiwan: J-L Huang*, Department of Pediatrics, Chang Gung Memorial Hospital, New Taipei Municipal TuChen Hospital, and Chang Gung University (Taipei); Thailand: P Vichyanond*, Mahidol University (Bangkok).

* National Coordinators

† Deceased

ISAAC Phase Three National Co-ordinators not named above: Chile: V. Aguirre, University of Santiago de Chile (USACH), Santiago, Chile; Mexico: M Baeza-Bacab, University Autónoma de Yucatán, Yucatán; Sudan: A El Sony Epidemiological Laboratory for Public Health and Research, Khartoum; Syrian Arab Republic: S Mohammad, Tishreen University, Lattakia.

ISAAC Phase One Principal Investigators: Chile: E Cortéz, Universidad de Santiago de Chile (USACH), Santiago, Chile (South Santiago); Costa Rica: ME Soto-Quirós*, University of Costa Rica (Costa Rica); Greece: CH Gratziou*, National Kapodistrian University of Athens (Athens); India: L Kumar †, Department of Pediatrics (Chandigarh); TU Sukumaran, PIMS Thiruvalla (Kottayam); K Chopra, Maulana Azad Medical College (New Delhi [7]); NM Hanumante, Bharati Vidyapeeth Medical College (Pune); New Zealand: MI Asher*, Department of Paediatrics: Child and Youth Health, University of Auckland (Auckland); Nigeria: BO Onadeko, (Ibadan); South Africa: H Nelson, Horsett Hospital (Cape Town); Spain: AD Rubio, Urgencias de Pediatria. Pabellon Makua, Bilbao, Spain (Bilbao); L García-Marcos*, Pediatric Allergy and Pulmonology Units, Virgen de la Arrixaca University Children's Hospital, University of Murcia and IMIB Bioresearch Institute, Murcia; and ARADyAL Allergy Network, Edificio Departamental-Laib, Murcia Spain (Cartagena); Taiwan: K-H Hsieh †, Chang Gung Children's Hospital (Taipei); Thailand: P Vichyanond*, Mahidol University (Bangkok).

* National Coordinators

† Deceased

ISAAC Phase One National Co-ordinators not named above: Chile: J Mallol, University of Santiago de Chile (USACH), Santiago, Chile; India: J Shah, Jaslok Hospital & Research Centre, Mumbai.

Web Figure 1

Bland Altman plots which examined, for current wheeze, the relationship between change in prevalence and average prevalence between time points for both age groups.

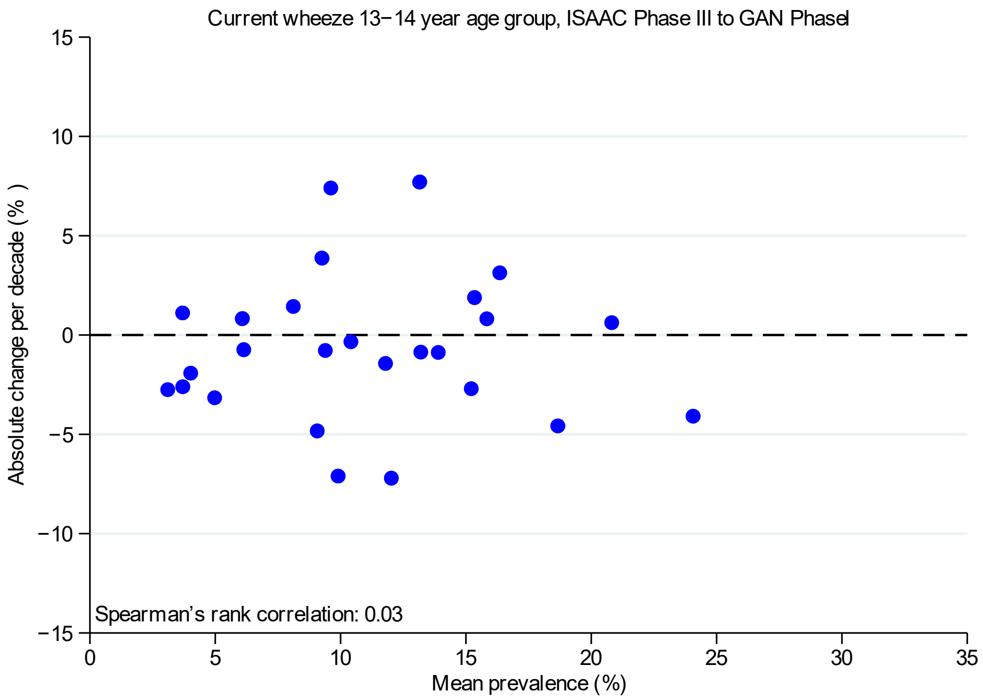
Current wheeze 13−14 year age group, ISAAC Phase III to GAN PhaseI
Absolute change per decade (%)
Mean prevalence (%)
Spearman's rank correlation: 0.03
15
10
5
0
−5
−10
−15
0
5
10
15
20
25
30
35

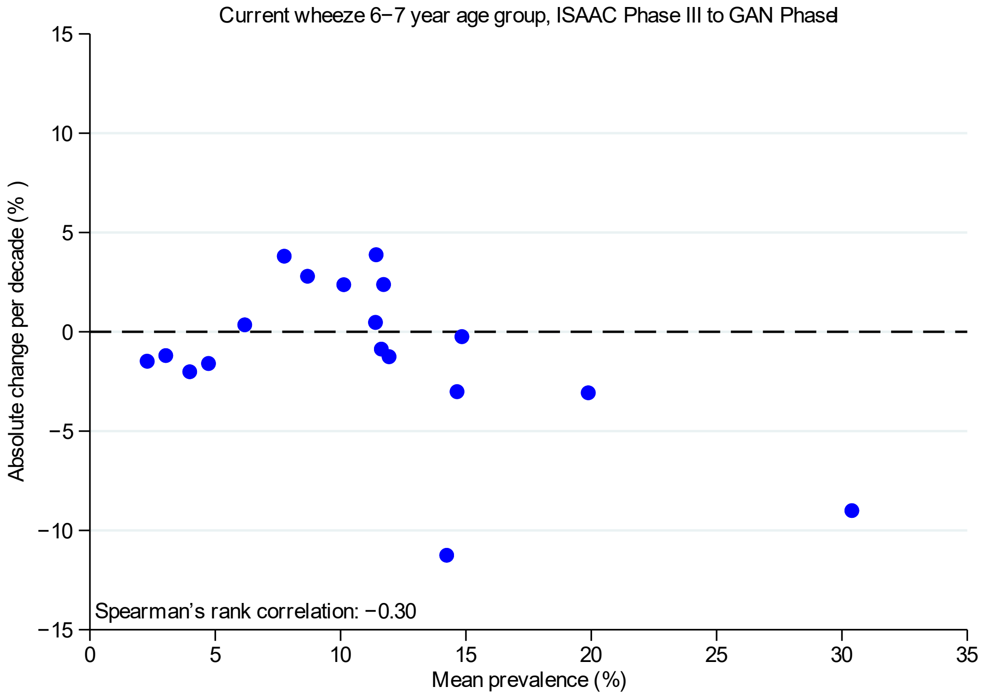
Current wheeze 6−7 year age group, ISAAC Phase III to GAN PhaseI
Absolute change per decade (%)
Mean prevalence (%)
Spearman's rank correlation: −0.30
15
10
5
0
−5
−10
−15
0
5
10
15
20
25
30
35

Web Figure 2

World map of centres, for 13-14 year olds (adolescents) showing changes in prevalence per year expressed as standard error (SE) per year, with dark blue triangle = prevalence reduced by ≥2 SE, light blue triangle = prevalence reduced by ≥1SE, green square = little change in prevalence <1 SE per year, light red triangle = prevalence increased by ≥1 SE, dark red triangle = prevalence increased by ≥2 SE: for current wheeze (2a), severe asthma symptoms (2b) and asthma ever (2c). Athens, included here, undertook ISAAC Phase I but not ISAAC Phase III.

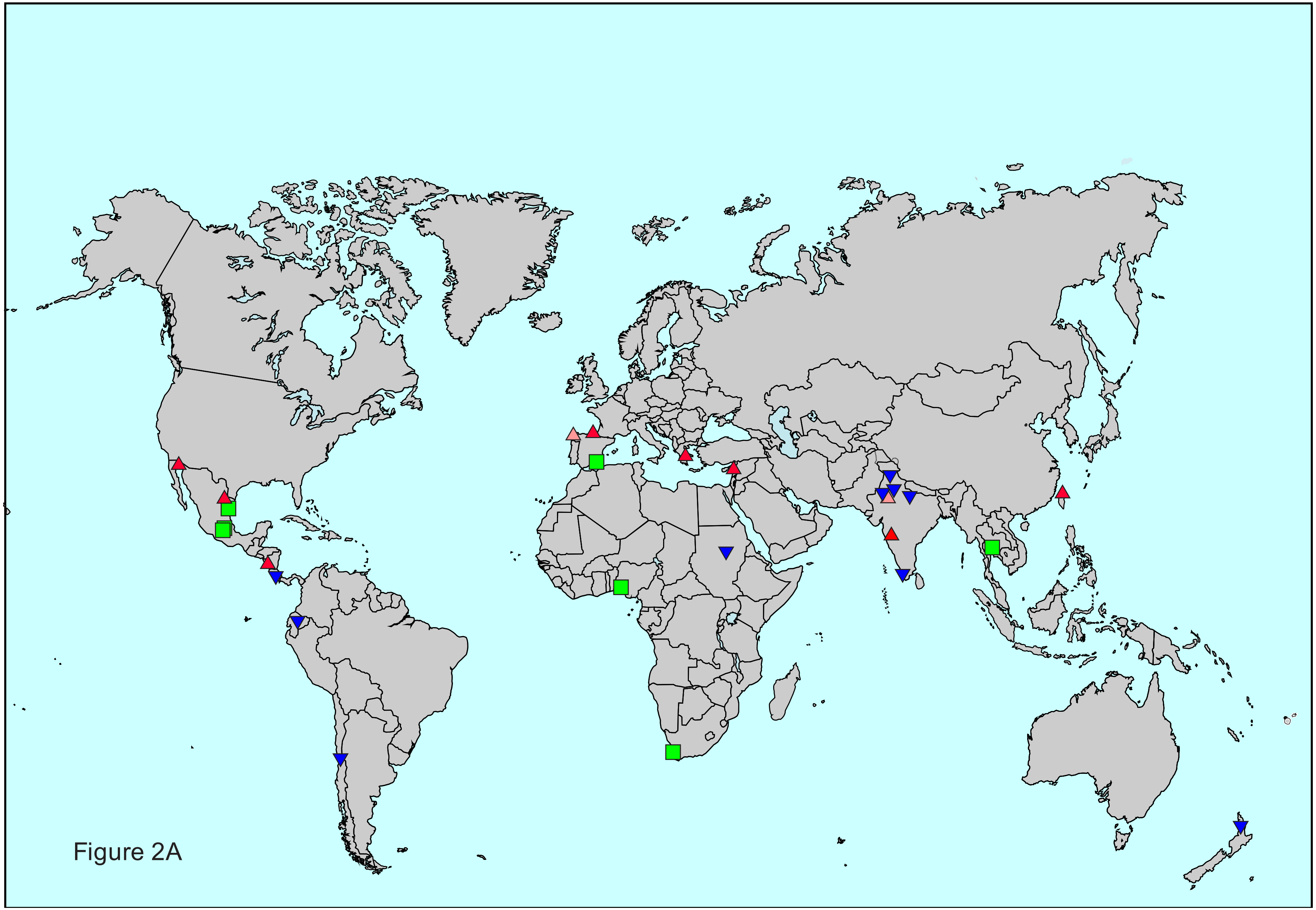

Figure 2A

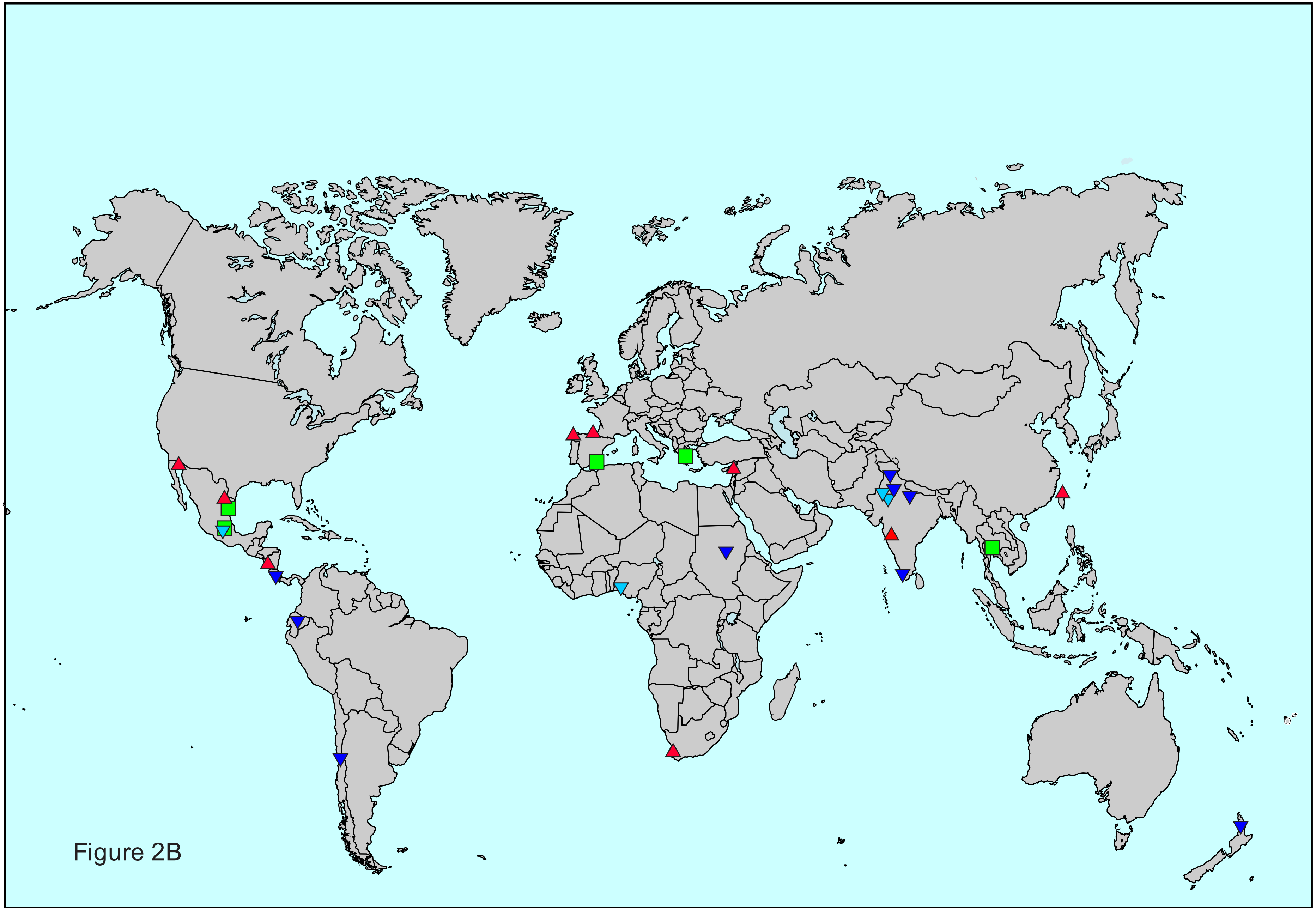
Figure 2B

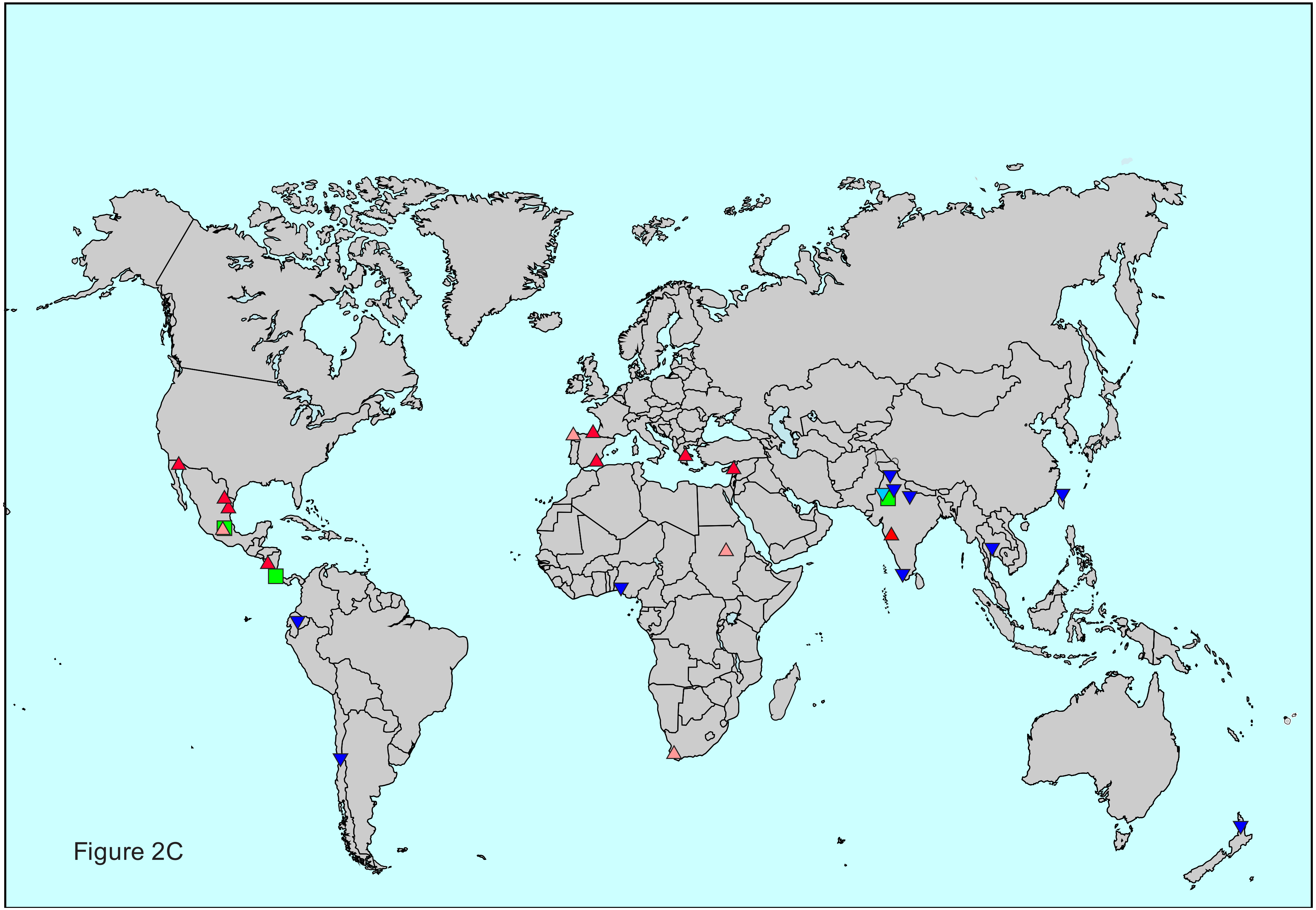
Figure 2C

Web Figure 3

World map of centres, for 6-7 year olds (children) showing changes in prevalence per year expressed as standard error (SE) per year, with dark blue triangle = prevalence reduced by ≥2 SE, light blue triangle = prevalence reduced by ≥1SE, green square = little change in prevalence <1 SE per year, light red triangle = prevalence increased by ≥1 SE, dark red triangle = prevalence increased by ≥2 SE: for current wheeze (3a), severe asthma symptoms(3b) and asthma ever (3c).

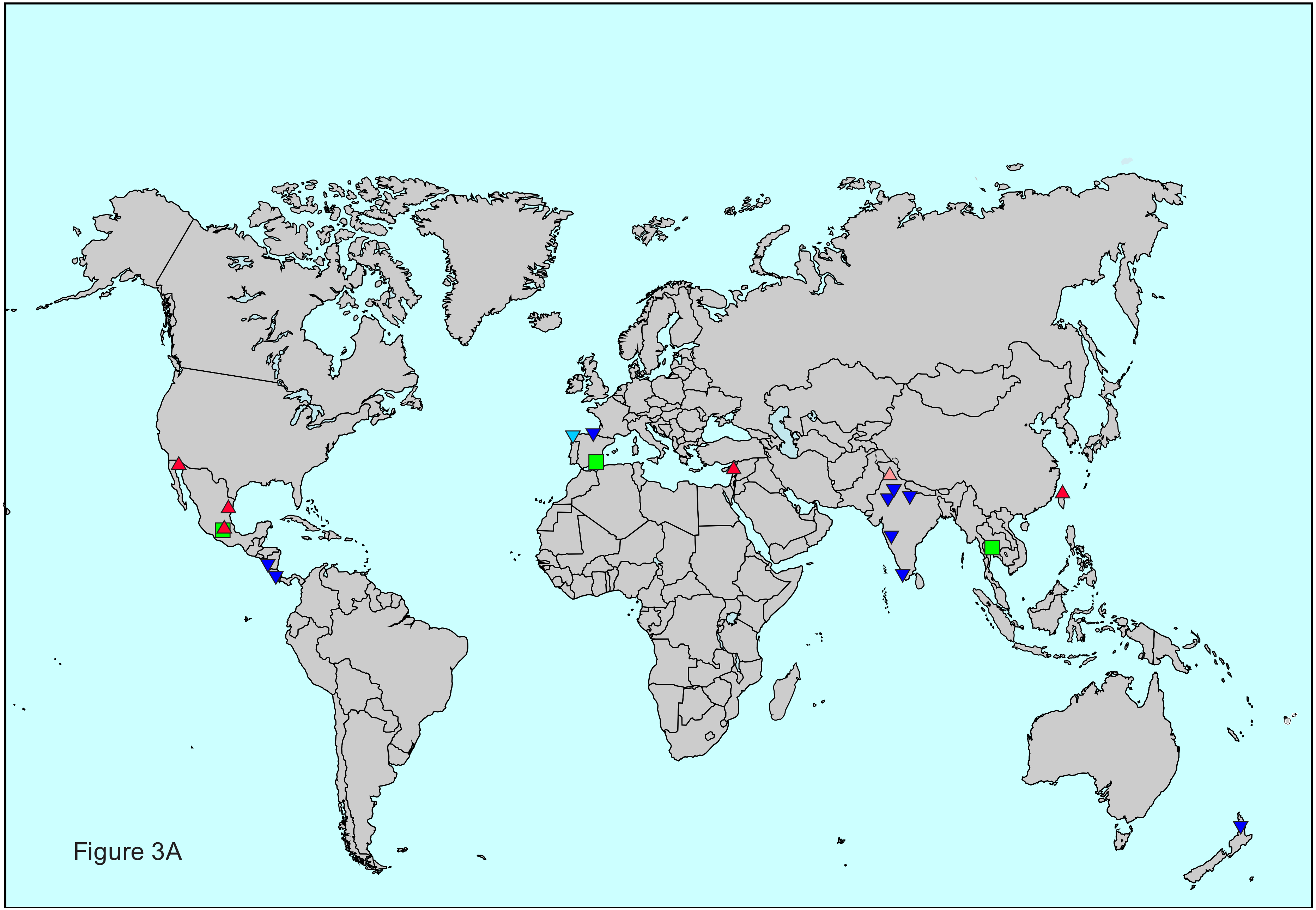
Figure 3A

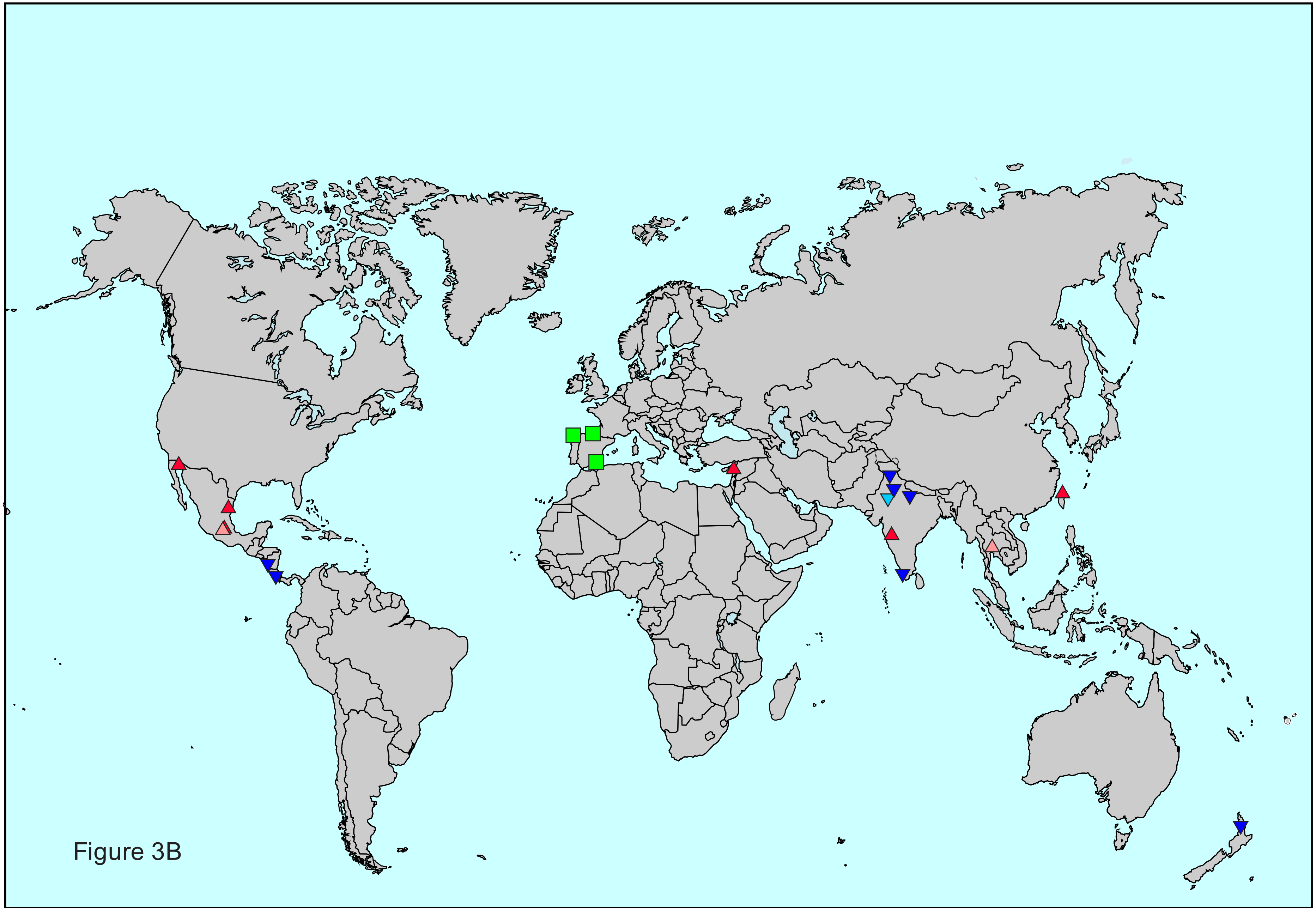
Figure 3B

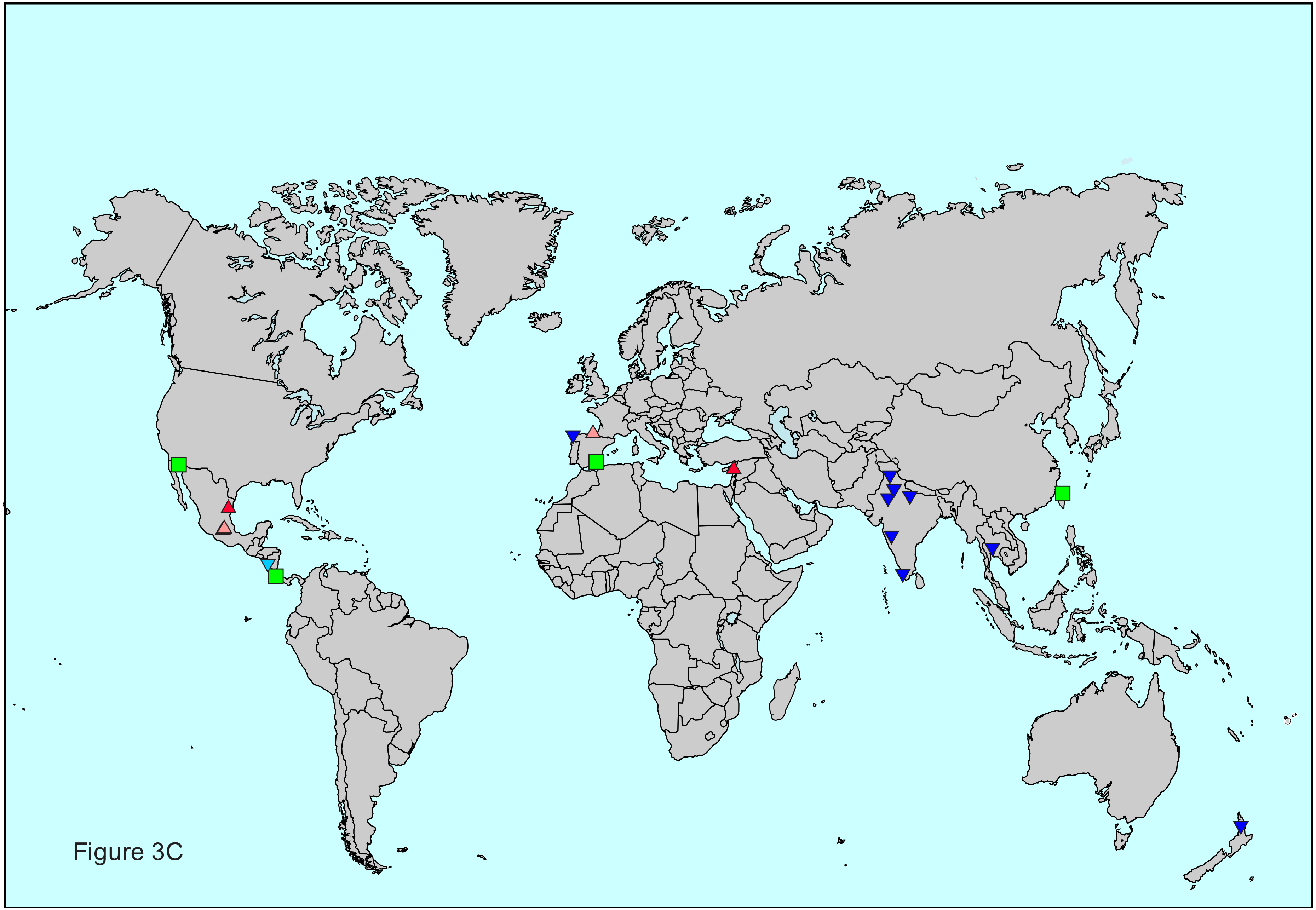

Figure 3C
